# OCT and OCT Angiography Offer New Insights and Opportunities in Schizophrenia Research and Treatment

**DOI:** 10.3389/fdgth.2022.836851

**Published:** 2022-02-18

**Authors:** Kyle M. Green, Joy J. Choi, Rajeev S. Ramchandran, Steven M. Silverstein

**Affiliations:** ^1^Department of Ophthalmology, University of Rochester Medical Center, Rochester, NY, United States; ^2^Department of Psychiatry, University of Rochester Medical Center, Rochester, NY, United States; ^3^Department of Public Health Sciences, University of Rochester Medical Center, Rochester, NY, United States; ^4^Department of Neuroscience, University of Rochester Medical Center, Rochester, NY, United States; ^5^Center for Visual Science, University of Rochester, Rochester, NY, United States

**Keywords:** schizophrenia, OCT, angiography, retina, biomarker

## Abstract

The human retina and retinal imaging technologies continue to increasingly gain the attention of schizophrenia researchers. With the same embryologic origin as the brain, the retina offers a window into neurovascular changes that may underlie disease. Recently, two technologies that have already revolutionized the field of ophthalmology, optical coherence tomography (OCT), and a functional extension of this, optical coherence tomography angiography (OCTA), have gained traction. Together, these non-invasive technologies allow for microscopic imaging of both structural and vascular features of the retina. With ease of use and no side effects, these devices are likely to prove powerful digital health tools in the study and treatment of schizophrenia. They may also prove key to discovering disease relevant biomarkers that underly neurodevelopmental and neurodegenerative aspects of conditions such as schizophrenia.

## Introduction

Schizophrenia is a devastating and complex disease, carrying a lifetime morbid risk of ~0.7% (1). Equally complex as the disease itself is its etiology, comprising a multitude of genetic and environmental factors. Genome wide association studies have identified hundreds of involved genetic loci. Other studies have consistently indicated that schizophrenia involves a neurodevelopmental component, with motor, cognitive, and behavioral changes often apparent many years before the emergence of psychotic symptoms (2, 3). Moreover, environmental influences can make significant contributions to the expression and timing of schizophrenia due to epigenetic and other biological changes (e.g., hippocampal atrophy due to childhood trauma mediated by HPA axis dysregulation) (4, 5).

Interestingly, results from genetic association studies that evaluate schizophrenia-associated genes indicate there is an overrepresentation of genes related to vascular function (6), and a growing number of researchers are considering a possible central role of vascular dysfunction in the etiology of schizophrenia. For instance, Hanson and Gottesman proposed that abnormalities in blood flow may lead to neuronal-glial dysfunction which ultimately leads to psychopathology, challenging more traditional thinking of vascular dysfunction as secondary (7).

Cardiovascular disease, a sequela of vascular dysfunction, is a major contributing factor to shortened life expectancy by 10–20 years in individuals with schizophrenia, accounting for up to 40% of natural death in this population (8, 9). The traditional thinking of vascular dysfunction as a secondary finding in schizophrenia has resulted in research focusing on and calling for secondary prevention of cardiovascular disease in this population (10–12). However, the newer conceptualization of vascular dysfunction as a central piece to the pathophysiology of schizophrenia opens up a new possibility for primary prevention of cardiovascular disease with early screening of vascular dysfunction in individuals with schizophrenia, or even those at risk of the disease (13).

Schizophrenia may be best described as a neurovascular developmental and degenerative disease (14). Therefore, an approach to characterize and measure changes in multiple involved tissues throughout the disease course is paramount to develop new understanding. As a relatively well understood neurovascular tissue in the human eye, the human retina has—with good reason—rapidly gained attention from schizophrenia researchers. There are a number of reasons for this. Firstly, it is derived from the same embryologic origin as the brain. Secondly, unlike any other neurovascular tissues in the body, it is readily observable with non-invasive imaging techniques. Thirdly, it has a well-defined laminar structure and vascular supply that can be imaged with a revolutionary imaging modality called optical coherence tomography (OCT) and a functional extension of this modality, optical coherence tomography angiography (OCTA). OCT is a non-invasive imaging technology that uses low-coherence interferometry to generate high resolution structural images of the retina. By comparing repeated structural images throughout the retina, OCTA is able to detect areas of motion that reflect the movement of red blood cells, and subsequently generate high resolution angiograms.

**Figure 1:**
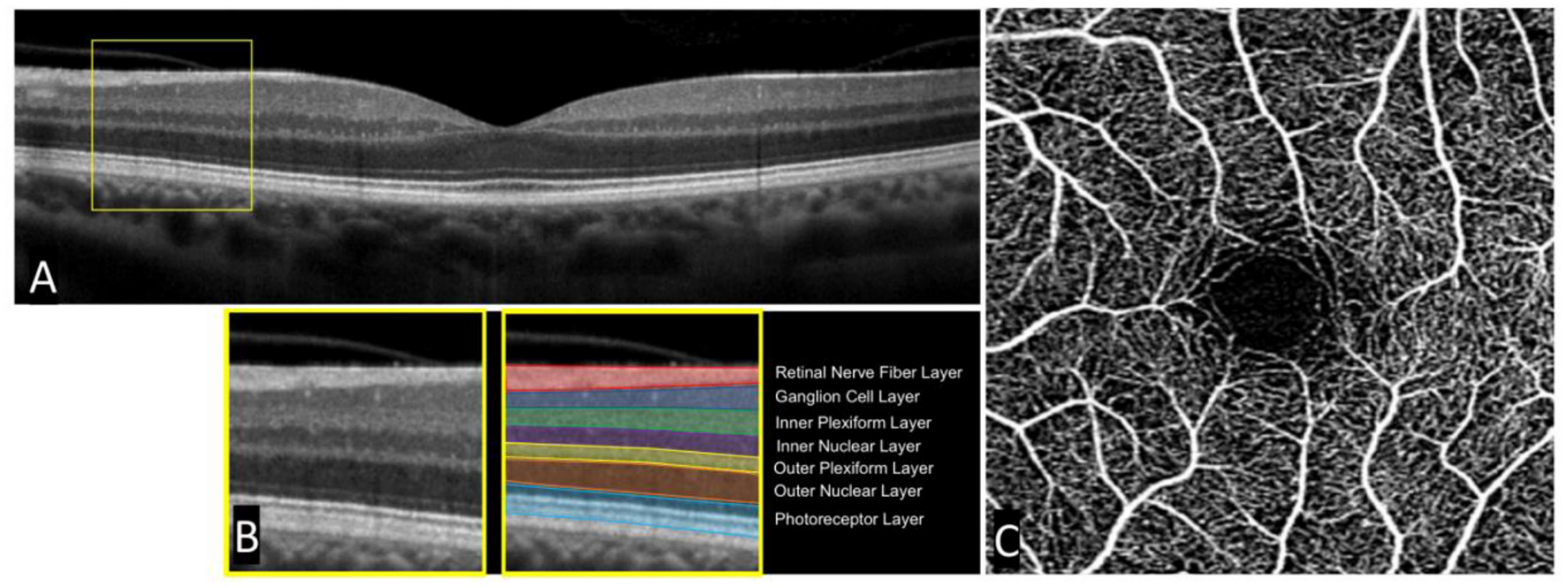
Single cross-sectional OCT scan and accompanying OCT angiogram in a healthy subject. A standard cross-sectional OCT scan of a healthy human retina **(A)**. Individual layers can be seen in an enlarged portion of the OCT scan **(B)**. A standard OCT angiogram of a healthy patient with a central foveal avascular zone **(C)**.

The development of OCT revolutionized the field of ophthalmology and retinal research, affording clinicians and researchers alike images of the human retina with microscopic detail. Though with completely different mechanisms that generate the images, OCT scans of the retina can be described as analogous to the better known computed tomography scans or ‘CT scans' of the brain. Both provide cross-sectional images of the retina and brain respectively, albeit with different resolutions, that can be analyzed. Compared to CT (and MRI), OCT provides significantly better resolution of retinal tissue at ~5–20 μm axially (15). Similarly, OCTA scans of the retina can be described as analogous to CT angiography scans of the brain, providing detailed angiograms of the vasculature. OCTA is performed on the same device as OCT, and is able to identify blood vessels by comparing repeated OCT scans throughout the retina and extracting signals of motion (reflecting the motion of red blood cells), which are used to define the location, length, and width of blood vessels. Unlike CT and CT angiography however, OCT and OCTA do not use radiation, nor any other form of contrast, and require a fraction of the time to perform. They are also more portable and ubiquitous, being present in nearly all modern eye clinics. Impressively, modern OCT imaging is completely safe with no known side effects, and can produce high quality and highly reliable (e.g., intraclass correlation > 0.99) images in a matter of seconds (16). Furthermore, OCT and OCTA provide microscopic resolution with imaging of distinct cellular layers in the retina and microvasculature down to the level of the capillaries. A single cross section of an OCT scan and full-thickness OCTA are seen in Figure 1.

Now with the power to rapidly and non-invasively image microscopic *in-vivo* changes in neurovascular tissue, researchers are primed to explore a new realm of opportunities in the understanding of schizophrenia. Here, we briefly summarize the current state of research in schizophrenia using these two imaging modalities, OCT and OCTA, and further highlight the immense opportunity they present for future study.

## Utility of OCT

The true power of OCT lies in its resolution, able to detail individual cellular retinal layers and measure changes over time on the scale of micrometers. Changes in retinal structure may serve as a surrogate marker for similar changes in the brain. Many studies have now demonstrated similar changes in structure and function in retina and brain in both neurologic disease and normal aging [reviewed in (17)].

Comprehensive review of specific OCT findings in schizophrenia has been performed previously, but we will briefly discuss several key issues here. A main focus of OCT research has been the measured thickness of the retinal nerve fiber layer (RNFL), as well as the total macular volume and thickness. The RNFL is comprised of long axons that originate from superficial ganglion cells scattered throughout the retina. This layer can be easily seen in Figure 1. Together, these axons project to a common point in the retina where they then exit the back of the eye to form the optic nerve and do not synapse until reaching the lateral geniculate nucleus in the thalamus. RNFL thickness is clinically relevant in a number of ophthalmologic diseases, most notably glaucoma, where disease progression is measured over time by a reduction in total RNFL thickness. Interestingly, a number of studies have demonstrated reductions in RNFL thickness in schizophrenia compared to healthy controls and others have similarly demonstrated a reduction in macular volume and thickness (17).

The pathophysiology of these changes in schizophrenia is poorly understood. Acaso et al. (18) suggest that neuroinflammation associated with acute episodes of psychosis may be responsible. Structural changes in the retina likely reflect similar changes that are observable in the brain. In one study that assessed patients with first-episode schizophrenia at baseline and at 3 years, there were reductions in both retinal thickness as seen on OCT and in visual cortex grey matter volume as measured on MRI (19).

Co-morbid conditions that are disproportionately common in schizophrenia patients threaten to confound studies that use OCT. For one, patients with schizophrenia have a higher incidence of both diabetes and hypertension (20), both of which are associated with retinal thinning (21, 22). However, patients with schizophrenia have demonstrated enlarged “cup to disc” ratios in their optic nerves, an ophthalmologic finding that results from loss of the retinal nerve fiber layer, even when controlling for these factors (23), and multiple studies that have excluded schizophrenia patients with comorbid medical conditions known to affect the retina have nevertheless demonstrated RNFL and macula volume and thickness reductions (17).

The future of these devices in schizophrenia research is bright, especially as OCT devices continue to rapidly evolve. For example, compared to the Zeiss Cirrus 5000 model which performs 27,000 axial scans per second, the Cirrus 6000 performs 100,000 scans per second and was released only a few years later. In the coming years, OCT is primed to offer even higher resolution images and will likely be able to detect even more subtle changes in retinal architecture in early disease. Though not yet readily available for human study, one technology called “adaptive optics OCT” aims to allow for imaging of the retina down to the level of individual cells. This would allow for more accurate quantification of cell density and possibly connectivity which likely differ in disease states compared to healthy controls. It may also provide new biomarkers not yet conceived and help clarify aspects of altered retinal computation (24). With numerous possibilities on the horizon, the stage is set for OCT to revolutionize our understanding of many neurologic diseases, schizophrenia among them.

## Utility of OCTA

As discussed previously, the theory that vascular dysfunction plays a primary role in the development and progression of schizophrenia continues to gain traction. This dysfunction is likely to involve changes in the smallest caliber vessels that supply neural tissue, capillaries. Indeed, the diagnosis of schizophrenia has been found to correlate with impaired microvascular function (25). Changes have also been found in larger caliber vessels in the retina. Using fundus photography, one study found that patients with schizophrenia had significantly narrower arterioles and wider venules compared to healthy controls (26). Since becoming commercially available in 2014, OCTA has allowed researchers to assess vessels of all caliber in the retina with a high degree of precision.

Only very recently, studies including our own have explored various microvascular features in schizophrenia using OCTA. Previous studies pioneered the development of methods to quantify the density and morphology of vasculature based on images acquired using this device (27). A commonly used metric simply called “vessel density” (VD) is defined as the proportion of an angiogram that is filled with vessels compared to avascular background. The higher the proportion, the higher the density. This metric amongst others has been used to reliably characterize vascular changes in well understood vascular diseases, such as diabetic retinopathy (28). One limitation of assessing vessel density is the variation in the size of vessels that fall within a given imaging window. If an image happens to have several larger vessels that fall in the imaging window in lieu of capillaries, estimates of capillary density would be artificially inflated. This can be overcome in part by using a metric called “skeleton vessel density,” which is calculated by adjusting all vessels in an image to an equal diameter. This essentially provides a total estimate of vessel length, not confounded by width, in the image. Another common measure is the size of an avascular central area of the retina referred to as the “foveal avascular zone” or FAZ. Without obstructing vessels, photoreceptors in the FAZ are able to receive unimpeded light resulting in high resolution central vision. An enlarged FAZ has been long established to be present in retinal vascular diseases, and reflects a loss of retinal vasculature (29).

**Figure 2:**
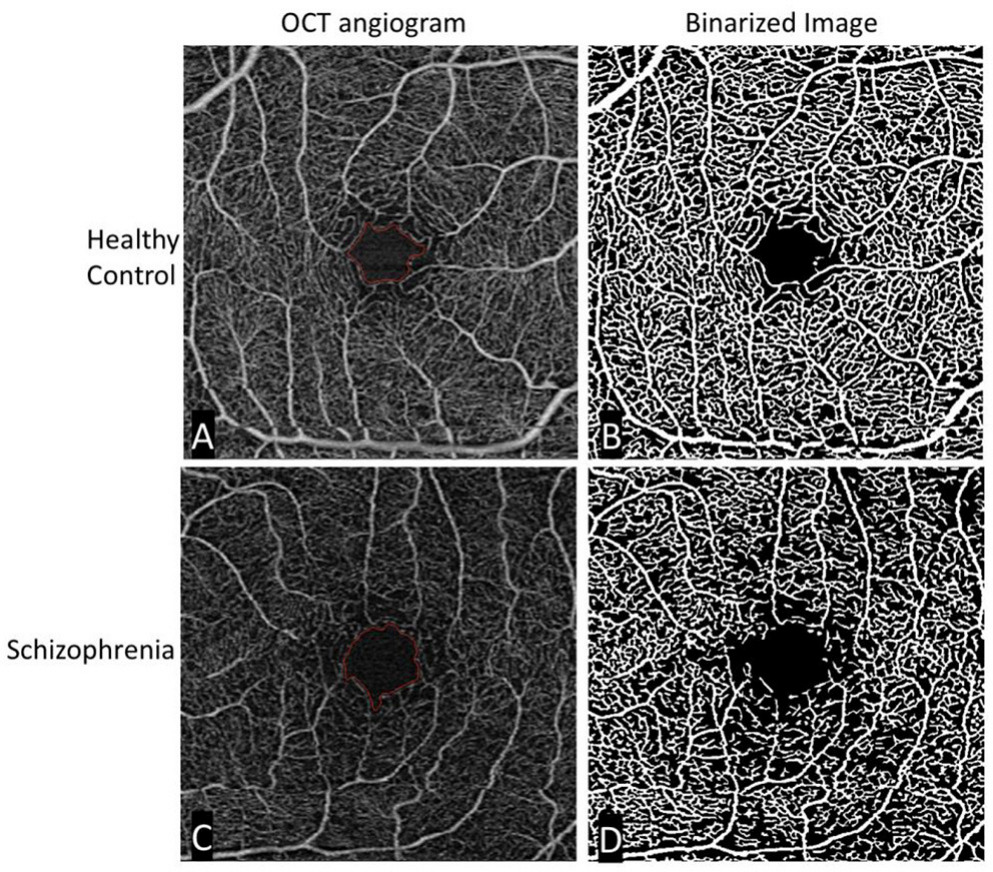
Original and binarized OCT angiograms of a patient with schizophrenia and a healthy control. A standard OCT angiogram of a healthy eye is seen in **(A)**. Vessels are seen in white, with larger arterioles and smaller capillaries represented in the 3 × 3 mm^2^ window. The foveal avascular zone is delineated in red. **(B)** Binarized version of the same angiogram, allowing for easier qualitative and quantitative assessment of vascular density. The bottom row **(C,D)** shows similar images of a patient with schizophrenia. Compared to the healthy control, there is an enlargement in the foveal avascular zone and a decrease in vessel density most easily appreciated in the binarized image.

The limited number of published studies using OCTA in schizophrenia can be briefly summarized. Our study found that individuals with schizophrenia demonstrated reductions in microvascular density and an increase in foveal avascular zone area compared to controls (30). An illustration of this can be seen in Figure 2. Both measures reflect a measurable loss of capillaries perfusing the retina. Koman-Wierdak et al. (31) similarly found that individuals with schizophrenia had reduced vascular density in the retina compared to controls, specifically in the deep capillary plexus. A more recent study found an increase in superficial skeleton vessel density, choriocapillaris skeleton vessel density, and choriocapillaris vessel density in individuals with schizophrenia compared to controls (32). When looking at peripapillary vascular density (vascular density surrounding the optic nerve), Budakoglu et al. (33) found that individuals with schizophrenia had reduced density in one quadrant compared to controls.

These studies all demonstrate measurable differences in vascular density between individuals with schizophrenia and healthy controls. Interestingly, Bannai et al.'s finding of increased superficial vascular density in schizophrenia, albeit significant only in the central sub-region of the macula in right eyes, contrasts with the reduction seen in other studies. This may suggest layer specific changes in the disease course over time, or regional differences in the retina. Similar differences have also been noted in other diseases using OCTA, such as multiple sclerosis (34). One group provides a possible explanation for layer-specific differences, positing that loss of vasculature in one layer could lead to compensatory angiogenesis in another, thereby increasing vascular density (35).

With so few studies, additional data is needed. While much attention has been paid to vascular density, metrics that assess vascular morphology may also prove useful. This includes vessel diameter index, which assesses average vessel diameter, as well as vessel complexity, a measure of tortuosity (28). “Flux” is another promising metric, which reflects the relative number of red blood cells moving through a vessel segment per unit of time, thereby quantifying physiological blood flow at the capillary level (36).

## Discussion

Given the features described of these imaging modalities, OCT and OCTA individually present unique opportunities to explore both the neuroanatomical and microvascular characteristics of the retina in schizophrenia. Lacking in current research however are studies that aim to take advantage of their combined use, as well as their ability to aid in longitudinal analysis. Additionally, larger sample sizes are needed to provide better estimates of effect size, and to more clearly link differences in effects to aspects of illness heterogeneity, duration, and phase (e.g., acute vs. remitted). Used together in a longitudinal study, these technologies may help elucidate a temporal relationship between observed microvascular changes and neuroanatomical changes in the retina. For instance, if vascular loss were found to precede loss of neural tissue, this may lend evidence toward a more causal relationship. As OCT and OCTA can be performed on the same device, such a research aim is readily feasible, and data from one first episode psychosis sample provides preliminary evidence for vascular changes that precede neural changes (30, 37). Moreover, OCTA has been used previously to detect subtle vascular changes in such as radiation retinopathy over time periods as short as 6 months (38). In one study that imaged patients with multiple sclerosis with OCTA at baseline and 1 year, patients with a history of optic neuritis were found to have a significant decrease in volumetric vessel density (39). Therefore, even observations over a humble time period in the setting of an extended disease course may yield fruitful results.

Additional opportunities exist in using these modalities to explore any acute retinal changes in subjects with schizophrenia following episodes of psychosis. Albeit challenging to obtain, pre and post episode imaging of individuals with first-episode psychosis would be particularly interesting. Also of interest are the effects of antipsychotic medications, which previous studies have identified to have a relationship with structural changes in both the retina and grey matter cortical volume (19, 40), although the retinal effects are generally rarer than disease-related retinal changes. Therefore, OCT and OCTA could be used for both short and long-term assessment of retinal alterations in individuals with schizophrenia taking antipsychotic medications. A comparison with individuals on antipsychotic medications without schizophrenia may help to parse disease from medication effects, despite some shared genetic basis between conditions such as schizophrenia, bipolar disorder, and major depression, as has been demonstrated in the case of retinal neural functioning as assessed *via* electroretinography (41, 42). An additional method of separating disease from medication effects would be to determine if schizophrenia-like findings are observed in unaffected first-degree relatives of people with a psychotic disorder (43), and/or in other people considered to be at high risk for the disorder (e.g., those considered to be at clinical high-risk but who do not yet meet full syndromal criteria and who have not yet received antipsychotic medication) (44). Comparing medicated and unmedicated patients can also be useful, although these populations typically differ in terms of illness history and severity.

One “holy grail” in clinical schizophrenia research is the identification of biomarkers of early disease. Biomarkers may include already well-recognized findings found in individuals with schizophrenia compared to healthy controls, namely changes in capillary density and anatomic changes in retinal cell layers. Lending credence to this idea, one study of older Latinx adults observed impaired retinal capillary perfusion using OCTA in the presence of only minimal cognitive impairment, non-specific to any one disease (45). Instead of using these biomarkers to make definitive clinical diagnoses, they are likely to aid clinicians and researchers in better assessing risk of disease development and progression. In terms of disease development, by characterizing a “retinal profile” in individuals with schizophrenia, and then determining the extent to which people at genetic or clinical high risk for schizophrenia begin to be characterized by this profile, better prediction of development of a psychotic disorder may be achieved. Already, findings from electroretinography support this approach (42, 46). Below, we discuss the utility of OCTA for identifying those schizophrenia patients at greatest risk for progressive neurodegeneration and cognitive and functional decline.

One promising aspect of OCT technology that may dramatically increase the amount of available data, and clinical utility, is its emerging portability. Options for at home self-administered OCT imaging are already under investigation (47, 48). The clinical goals of at home testing include early detection of disease through screening, closer monitoring of active disease progression, and a reduction in the number of necessary in-person clinical visits. An increase in routine imaging could offer opportunities for longitudinal OCT studies, which are challenging to perform.

Another frontier in schizophrenia research is the application of artificial intelligence, namely machine learning, to identify and analyze the volumetric data obtained in OCT and OCTA scans. Recent studies have been able to develop a predictive model for mild cognitive impairment (49) and Alzheimer's disease through the use of machine learning applied to vascular feature extraction from fundus images (50). Compared to fundus images where smaller vessels can be challenging to appreciate, OCT angiograms can provide even greater vascular detail at the capillary level. Furthermore, unlike 2-D fundus photos, OCT scans provide volumetric data. Indeed, recent studies have utilized machine learning, as well as deep-learning approaches (a specific class of machine learning), to classify different retinopathies (51). Artificial intelligence may be similarly used to first identify biomarkers with higher specificity to schizophrenia, with subsequent applications to detect and even predict emerging schizophrenia. However, diagnostic specificity is not necessary for OCT data to be useful in the clinical management of people with, or at risk for, schizophrenia, for several reasons. First, the DSM diagnostic criteria for schizophrenia have undergone changes four times in the past 42 years, and this is likely to continue due to the heterogeneity of the syndrome, the less than adequate reliability for assessing diagnostic criteria, and the lack of an understanding of its etiology (52–54). Second, in neurology there is no evidence yet for specificity of OCT and OCTA findings for conditions such as multiple sclerosis, Parkinson's disease, Alzheimer's disease, and Huntington's disease, even though findings are reliably associated with each disease and with specific illness features. However, as noted above, OCT and OCTA data provide value by indicating, cross-sectionally, evidence of CNS degeneration, and longitudinally, evidence of further disease progression, including cognitive decline, that can indicate the need for neuroprotective interventions (55). In schizophrenia, where the extent of cognitive decline and deterioration in functioning throughout adulthood is highly variable (56–58), brief, reliable, valid, non-invasive, inexpensive, and (in some cases) portable methods such as OCT and OCTA can serve an important and unique functioning in patient monitoring and treatment planning efforts, regardless of the extent to which they can serve as diagnostic aids.

Lastly, the increasing speed and therefore decreasing acquisition time of OCT devices may help to improve data quality. Some patients, especially those who have difficulty sitting in a still position and fixating their gaze at one point, often have difficulty sitting through the brief acquisition period. As a result, the images produced may have artifacts that interfere with calculation of the discussed metrics, such as vessel density. With faster acquisition times, as well as further development of adaptive optics methods, this limitation may be overcome.

In conclusion, the human retina is likely to remain a prime target for researchers hoping to elucidate the complexities of schizophrenia. Moreover, there will undoubtedly be increasingly better resolution and speed, and advances in capacity (e.g., measurement of blood cell flow rate through retinal vessels) over the next few years. Likewise, methods to analyze data collected from these technologies are growing in their sophistication, and will potentially extract details that would otherwise escape traditional analysis. Perhaps most exciting, the study of schizophrenia using these technologies has only just begun. While OCT has already revolutionized the field of ophthalmology, it may be primed to do the same for the broader study of neurodegenerative and neurovascular disease. Thus, OCT and OCTA are leading candidates for portable, rapid, non-invasive digital health tools focused on disease-relevant biomarkers that can be used in cases of some of the most disabling neuropsychiatric conditions, including schizophrenia.

## Ethics Statement

The studies involving human participants were reviewed and approved by University of Rochester Research Subjects Review Board. The patients/participants provided their written informed consent to participate in this study.

## Author Contributions

KG, RR, JC, and SS contributed significantly to the development of this manuscript and including writing and intellectual input. All authors contributed to the article and approved the submitted version.

## Funding

Work on this paper was supported by a grant from the New York Fund for Innovation in Research and Scientific Talent (NYFIRST), *Center for Eye and Brain Health* to author SS, as well as an unrestricted grant from Research to Prevent Blindness to RR.

## Conflict of Interest

The authors declare that the research was conducted in the absence of any commercial or financial relationships that could be construed as a potential conflict of interest.

## Publisher's Note

All claims expressed in this article are solely those of the authors and do not necessarily represent those of their affiliated organizations, or those of the publisher, the editors and the reviewers. Any product that may be evaluated in this article, or claim that may be made by its manufacturer, is not guaranteed or endorsed by the publisher.

## References

[1] McGrath J Saha S Chant D Welham J. Schizophrenia: a concise overview of incidence, prevalence, and mortality. Epidemiol Rev. (2008) 30:67–76. 10.1093/epirev/mxn001 18480098

[2] Schenkel LS Silverstein SM. Dimensions of premorbid functioning in schizophrenia: a review of neuromotor, cognitive, social, and behavioral domains. Genet Soc Gen Psychol Monogr. (2004) 130:241–70. 10.3200/MONO.130.3.241-272 15819307

[3] Schiffman J Walker E Ekstrom M Schulsinger F Sorensen H Mednick S. Childhood videotaped social and neuromotor precursors of schizophrenia: a prospective investigation. Am J Psychiatry. (2004) 161:2021–7. 10.1176/appi.ajp.161.11.2021 15514402

[4] Pruessner M Cullen AE Aas M Walker EF. The neural diathesis-stress model of schizophrenia revisited: an update on recent findings considering illness stage and neurobiological and methodological complexities. Neurosci Biobehav Rev. (2017) 73:191–218. 10.1016/j.neubiorev.2016.12.013 27993603

[5] Holtzman CW Trotman HD Goulding SM Ryan AT Macdonald AN Shapiro DI. Stress and neurodevelopmental processes in the emergence of psychosis. Neuroscience. (2013) 249:172–91. 10.1016/j.neuroscience.2012.12.017 23298853 PMC4140178

[6] Moises HW Wollschlager D Binder H. Functional genomics indicate that schizophrenia may be an adult vascular-ischemic disorder. Transl Psychiatry. (2015) 5:e616. 10.1038/tp.2015.103 26261884 PMC4564558

[7] Hanson DR Gottesman II. Theories of schizophrenia: a genetic-inflammatory-vascular synthesis. BMC Med Genet. (2005) 6:7. 10.1186/1471-2350-6-7 15707482 PMC554096

[8] Laursen TM Munk-Olsen T Vestergaard M. Life expectancy and cardiovascular mortality in persons with schizophrenia. Curr Opin Psychiatry. (2012) 25:83–8. 10.1097/YCO.0b013e32835035ca 22249081

[9] Sweeting J Duflou J Semsarian C. Postmortem analysis of cardiovascular deaths in schizophrenia: a 10-year review. Schizophr Res. (2013) 150:398–403. 10.1016/j.schres.2013.08.029 24028743

[10] Correll CU Robinson DG Schooler NR Brunette MF Mueser KT Rosenheck RA. Cardiometabolic risk in patients with first-episode schizophrenia spectrum disorders: baseline results from the RAISE-ETP study. JAMA Psychiatry. (2014) 71:1350–63. 10.1001/jamapsychiatry.2014.1314 25321337

[11] De Hert M Vancampfort D Correll CU Mercken V Peuskens J Sweers K. Guidelines for screening and monitoring of cardiometabolic risk in schizophrenia: systematic evaluation. Br J Psychiatry. (2011) 199:99–105. 10.1192/bjp.bp.110.084665 21804146

[12] Hennekens CH Hennekens AR Hollar D Casey DE. Schizophrenia and increased risks of cardiovascular disease. Am Heart J. (2005) 150:1115–21. 10.1016/j.ahj.2005.02.007 16338246

[13] Meier MH Hill ML Breitborde NJK. Retinal imaging: a new tool for studying underlying liability to cardiovascular disease in schizophrenia. Current Psychiatry Reviews. (2016) 12:326–34. 10.2174/1573400512666160927145417

[14] Ouellette J Lacoste B. From neurodevelopmental to neurodegenerative disorders: the vascular continuum. Front Aging Neurosci. (2021) 13. 10.3389/fnagi.2021.749026 34744690 PMC8570842

[15] Aumann S Donner S Fischer J Müller F. Optical Coherence Tomography (OCT): Principle and Technical Realization. In: Bille J., editor. High Resolution Imaging in Microscopy and Ophthalmology. Cham: Springer (2019). 32091846

[16] Wadhwani M Bali SJ Satyapal R Angmo D Sharma R Pandey V. Test-retest variability of retinal nerve fiber layer thickness and macular ganglion cell-inner plexiform layer thickness measurements using spectral-domain optical coherence tomography. J Glaucoma. (2015) 24:e109–15. 10.1097/IJG.0000000000000203 25517254

[17] Silverstein SM Fradkin SI Demmin DL. Schizophrenia and the retina: towards a 2020 perspective. Schizophr Res. (2020) 219:84–94. 10.1016/j.schres.2019.09.016 31708400 PMC7202990

[18] Ascaso FJ Rodriguez-Jimenez R Cabezón L López-Antón R Santabárbara J De la Cámara C. Retinal nerve fiber layer and macular thickness in patients with schizophrenia: influence of recent illness episodes. Psychiatry Res. (2015) 229:230–6. 10.1016/j.psychres.2015.07.028 26213374

[19] Zhuo C Ji F Xiao B Lin X Chen C Jiang D. Antipsychotic agent-induced deterioration of the visual system in first-episode untreated patients with schizophrenia maybe self-limited: findings from a secondary small sample follow-up study based on a pilot follow-up study. Psychiatry Res. (2020) 286:112906. 10.1016/j.psychres.2020.112906 32151847

[20] Nishanth KN Chadda RK Sood M Biswas A Lakshmy R. Physical comorbidity in schizophrenia & its correlates. Indian J Med Res. (2017) 146:281–4. 10.4103/ijmr.IJMR_1510_15 29265031 PMC5761040

[21] Sahin OZ Sahin SB Ayaz T Karadag Z Turkyilmaz K Aktas E. The impact of hypertension on retinal nerve fiber layer thickness and its association with carotid intima media thickness. Blood Press. (2015) 24:178–84. 10.3109/08037051.2014.1000562 25658169

[22] Choi JA Kim HW Kwon JW Shim YS Jee DH Yun JS. Early inner retinal thinning and cardiovascular autonomic dysfunction in type 2 diabetes. PLoS ONE. (2017) 12:e0174377. 10.1371/journal.pone.0174377 28334035 PMC5363937

[23] Silverstein SM Paterno D Cherneski L Green S. Optical coherence tomography indices of structural retinal pathology in schizophrenia. Psychol Med. (2018) 48:2023–33. 10.1017/S0033291717003555 29233210

[24] Schwartz GW. Retinal Computation. Amsterdam: Elsevier Inc. (2021).

[25] Vetter MW Martin BJ Fung M Pajevic M Anderson TJ Raedler TJ. Microvascular dysfunction in schizophrenia: a case-control study. NPJ Schizophr. (2015) 1:15023. 10.1038/npjschz.2015.23 27336034 PMC4849449

[26] Appaji A Nagendra B Chako DM Padmanabha A Hiremath CV Jacob A. Retinal vascular abnormalities in schizophrenia and bipolar disorder: a window to the brain. Bipolar Disord. (2019) 21:634–41. 10.1111/bdi.12779 31009139

[27] Chu Z Lin J Gao C Xin C Zhang Q Chen CL. Quantitative assessment of the retinal microvasculature using optical coherence tomography angiography. J Biomed Opt. (2016) 21:66008. 10.1117/1.JBO.21.6.066008 27286188 PMC4901200

[28] Kim AY Chu Z Shahidzadeh A Wang RK Puliafito CA Kashani AH. Quantifying microvascular density and morphology in diabetic retinopathy using spectral-domain optical coherence tomography angiography. Invest Ophthalmol Vis Sci. (2016) 57:362–70. 10.1167/iovs.15-18904 27409494 PMC4968771

[29] Mansour AM Schachat A Bodiford G Haymond R. Foveal avascular zone in diabetes mellitus. Retina. (1993) 13:125–8. 10.1097/00006982-199313020-00006 8337493

[30] Silverstein SM Lai A Green KM Crosta C Fradkin SI Ramchandran RS. Retinal microvasculature in schizophrenia. Eye Brain. (2021) 13:205–17. 10.2147/EB.S317186 34335068 PMC8318708

[31] Koman-Wierdak E Róg J Brzozowska A Toro MD Bonfiglio V Załuska-Ogryzek K. Analysis of the peripapillary and macular regions using OCT angiography in patients with schizophrenia and bipolar disorder. J Clin Med. (2021) 10(18). 10.3390/jcm10184131 34575242 PMC8472507

[32] Bannai D Adhan I Katz R Kim LA Keshavan M Miller JB. Quantifying retinal microvascular morphology in schizophrenia using swept-source optical coherence tomography angiography. Schizophr Bull. 2021. 10.1093/schbul/sbab111 PMC8781445 34554256

[33] Budakoglu O Ozdemir K Safak Y Sen E Taskale B. Retinal nerve fibre layer and peripapillary vascular density by optical coherence tomography angiography in schizophrenia. Clin Exp Optom. (2021) 104:788–94. 10.1080/08164622.2021.1878816 33689623

[34] Rogaczewska M Michalak S Stopa M. Macular vessel density differs in multiple sclerosis and neuromyelitis optica spectrum disorder: an optical coherence tomography angiography study. PLoS One. (2021) 16:e0253417. 10.1371/journal.pone.0253417 34138942 PMC8211193

[35] Jiang H Gameiro GR Liu Y Lin Y Hernandez J Deng Y. Visual function and disability are associated with increased retinal volumetric vessel density in patients with multiple sclerosis. Am J Ophthalmol. (2020) 213:34–45. 10.1016/j.ajo.2019.12.021 31926161 PMC7214204

[36] Abdolahi F Zhou X Ashimatey BS Chu Z Jiang X Wang RK. Optical coherence tomography angiography-derived flux as a measure of physiological changes in retinal capillary blood flow. Transl Vis Sci Technol. (2021) 10(9) 10.1167/tvst.10.9.5 34342607 PMC8340668

[37] Lai A Crosta C Loftin M Silverstein SM. Retinal structural alterations in chronic versus first episode schizophrenia spectrum disorders. Biomark Neuropsychiatry. (2020) 2:100013. 10.1016/j.bionps.2020.100013

[38] Green KM Toy BC Ashimatey BS Mustafi D Jennelle RL Astrahan MA. Quantifying subclinical and longitudinal microvascular changes following episcleral plaque brachytherapy using spectral domain-optical coherence tomography angiography. J Vitreoretin Dis. (2020) 4:499–508. 10.1177/2474126420936199 33409441 PMC7785061

[39] Chen Q Jiang H Delgado S Hernandez J Alba DE Gregori G. Longitudinal study of retinal structure, vascular, and neuronal function in patients with relapsing-remitting multiple sclerosis: 1-year follow-up. Transl Vis Sci Technol. (2021) 10:6. 10.1167/tvst.10.6.6 34111252 PMC8107487

[40] Guo F Zhu YQ Li C Wang XR Wang HN Liu WM. Gray matter volume changes following antipsychotic therapy in first-episode schizophrenia patients: a longitudinal voxel-based morphometric study. J Psychiatr Res. (2019) 116:126–32. 10.1016/j.jpsychires.2019.06.009 31233895

[41] Hébert M Mérette C Paccalet T Gagné AM Maziade M. Electroretinographic anomalies in medicated and drug free patients with major depression: tagging the developmental roots of major psychiatric disorders. Prog Neuropsychopharmacol Biol Psychiatry. (2017) 75:10–5. 10.1016/j.pnpbp.2016.12.002 28007463

[42] Hébert M Mérette C Gagné AM Paccalet T Moreau I Lavoie J. The electroretinogram may differentiate schizophrenia from bipolar disorder. Biol Psychiatry. (2020) 87:263–70. 10.1016/j.biopsych.2019.06.014 31443935

[43] Gagné AM Moreau I St-Amour I Marquet P Maziade M. Retinal function anomalies in young offspring at genetic risk of schizophrenia and mood disorder: The meaning for the illness pathophysiology. Schizophr Res. (2020) 219:19–24. 10.1016/j.schres.2019.06.021 31320175

[44] Silverstein SM Thompson JL Gold JM Schiffman J Waltz JA Williams TF. Increased face detection responses on the mooney faces test in people at clinical high risk for psychosis. NPJ Schizophr. (2021) 7:26. 10.1038/s41537-021-00156-1 34001909 PMC8129098

[45] Ashimatey BS D'Orazio LM Ma SJ Jann K Jiang X Lu H. Lower retinal capillary density in minimal cognitive impairment among older Latinx adults. Alzheimers Dement. (2020) 12:e12071. 10.1002/dad2.12071 32875053 PMC7447879

[46] Moreau I Hébert M Maziade M Painchaud A Mérette C. (2022). The electroretinogram as a potential biomarker of psychosis in children at familial risk. Schizophrenia Bull Open. (in press) 10.1093/schizbullopen/sgac016 PMC11206048 39144760

[47] Ho AC Heier JS Holekamp NM Garfinkel RA Ladd B Awh CC. Real-world performance of a self-operated home monitoring system for early detection of neovascular age-related macular degeneration. J Clin Med. (2021) 10:1355. 10.3390/jcm10071355 33806058 PMC8036735

[48] Maloca P Hasler PW Barthelmes D Arnold P Matthias M Scholl HPN. Safety and feasibility of a novel sparse optical coherence tomography device for patient-delivered retina home monitoring. Transl Vis Sci Technol. (2018) 7:8. 10.1167/tvst.7.4.8 30050725 PMC6058910

[49] Zhang Q Li J Bian M He Q Shen Y Lan Y. Retinal imaging techniques based on machine learning models in recognition and prediction of mild cognitive impairment. Neuropsychiatr Dis Treat. (2021) 17:3267–81. 10.2147/NDT.S333833 34785897 PMC8579873

[50] Tian J Smith G Guo H Liu B Pan Z Wang Z. Modular machine learning for Alzheimer's disease classification from retinal vasculature. Sci Rep. (2021) 11:238. 10.1038/s41598-020-80312-2 33420208 PMC7794289

[51] Le D Son T Yao X. Machine learning in optical coherence tomography angiography. Exp Biol Med. (2021) 246:2170–83. 10.1007/978-3-030-65768-0 34279136 PMC8718258

[52] Wong SE. A critique of the diagnostic construct schizophrenia. Res Soc Work Pract. (2014) 2014:132–41. 10.1177/1049731513505152

[53] Cuthbert BN Insel TR. Toward new approaches to psychotic disorders: the NIMH research domain criteria project. Schizophr Bull. (2010) 36:1061–2. 10.1093/schbul/sbq108 20929969 PMC2963043

[54] Galderisi S Giordano GM. We are not ready to abandon the current schizophrenia construct, but should be prepared to do so. Schizophr Res. (2021) S0920–9964(21)00491-6. 10.1016/j.schres.2021.12.007. [Epub ahead of print]. 34924240

[55] Silverstein SM Demmin DL Schallek JB Fradkin SI. Measures of Retinal Structure and Function as Biomarkers in Neurology and Psychiatry. Biomark Neuropsychiatry. (2020) 2:100018. 10.1016/j.bionps.2020.100018

[56] Islam MA Habtewold TD van Es FD Quee PJ van den Heuvel ER Alizadeh BZ. Long-term cognitive trajectories and heterogeneity in patients with schizophrenia and their unaffected siblings. Acta Psychiatr Scand. (2018) 138:591–604. 10.1111/acps.12961 30242827 PMC6220939

[57] Shmukler AB Gurovich IY Agius M Zaytseva Y. Long-term trajectories of cognitive deficits in schizophrenia: a critical overview. Eur Psychiatry. (2015) 30:1002–10. 10.1016/j.eurpsy.2015.08.005 26516984

[58] Velthorst E Fett AJ Reichenberg A Perlman G van Os J Bromet EJ. The 20-year longitudinal trajectories of social functioning in individuals with psychotic disorders. Am J Psychiatry. (2017) 174:1075–85. 10.1176/appi.ajp.2016.15111419 27978770 PMC5474222

